# Mesenteric ischemia after capecitabine treatment in rectal cancer and resultant short bowel syndrome is not an absolute contraindication for radical oncological treatment

**DOI:** 10.2478/raon-2014-0024

**Published:** 2015-03-25

**Authors:** Ana Perpar, Erik Brecelj, Nada Rotovnik Kozjek, Franc Anderluh, Irena Oblak, Marija Skoblar Vidmar, Vaneja Velenik

**Affiliations:** 1 Department of Radiotherapy, Institute of Oncology Ljubljana, Ljubljana, Slovenia; 2 Department of Oncological Surgery, Institute of Oncology Ljubljana, Ljubljana, Slovenia; 3 Clinical Nutrition Unit, Institute of Oncology Ljubljana, Ljubljana, Slovenia

**Keywords:** rectal cancer, capecitabine, acute mesenteric ischemia, multiorgan failure, short bowel syndrome

## Abstract

**Background.:**

Thrombotic events, arterial or venous in origin, still remain a source of substantial morbidity and mortality in cancer patients. The propensity for their development in oncology patients is partially a consequence of the disease itself and partially a result of our attempts to treat it. One of the rarest and deadliest thromboembolic complications is arterial mesenteric ischemia. The high mortality rate is caused by its rarity and by its non-specific clinical presentation, both of which make early diagnosis and treatment difficult. Hence, most diagnoses and treatments occur late in the course of the disease. The issue survivors of arterial mesenteric ischemia may face is short bowel syndrome, which has become a chronic condition after the introduction of parenteral nutrition at home.

**Case report.:**

We present a 73-year-old rectal cancer patient who developed acute arterial mesenteric thrombosis at the beginning of the pre-operative radiochemotherapy. Almost the entire length of his small intestine, except for the proximal 50 cm of it, and the ascending colon had to be resected. After multiorgan failure his condition improved, and he was able to successfully complete radical treatment (preoperative radiotherapy and surgery) for the rectal carcinoma, despite developing short bowel syndrome (SBS) and being dependent upon home-based parenteral nutrition to fully cover his nutritional needs.

**Conclusions.:**

Mesenteric ischemia and resultant short bowel syndrome are not absolute contraindications for radical oncological treatment since such patients can still achieve long-term remission.

## Introduction

Standard treatment for locally advanced and/or node positive rectal cancer is neoadjuvant concomitant radiochemotherapy, surgery and adjuvant chemotherapy. All systemic therapy is 5-FU based.^1^,^2^

During treatment patients experience side effects, the most common of which are leukopenia, diarrhoea and proctitis, fatigue, nausea and vomiting, dermatitis, paraesthesia and hand-foot syndrome.^3^ Most patients require supportive measures and symptomatic therapy to complete treatment. Severe toxicity, *e.g*. thrombotic events or coronary vasospasm, is rare.^3^

We present a patient with locally advanced rectal cancer who developed severe and life-threatening complications during neoadjuvant treatment with mesenteric thrombosis and short bowel synrome. Good interdepartmental cooperation and multi-disciplinary treatment played a key role in the successful treatment first for mesenteric ischemia and then for rectal cancer as well.

To our knowledge, ours is the first case described wherein a patient with acute mesenteric ischemia was able to complete specific treatment for a malignant disease.

## Case report

A 73-year-old man presented with a 4-month history of bloody stools and weight loss. He had no previous relevant medical history. Magnetic resonance imaging (MRI) of the pelvis showed a T3N1 tumour of the rectum, 5 cm above the proximal margin of the anal sphincter.

Endoscopic biopsy confirmed a moderately differentiated adenocarcinoma. Abdominal ultra-sound and chest x-ray excluded the presence of distant metastases, setting the stage at IIIB.

The patient was referred to an oncology multi-disciplinary team, who made the decision to start pre-operative chemoradiotherapy after one cycle of induction chemotherapy with 2,500 mg/12h capecitabine within the framework of a national study. Written informed consent of patients was obtained for the treatments and for the scientific use of the clinical data according to Declarations of Helsinki.

After 10 days of chemotherapy the patient developed severe nausea, headaches, flushing and general weakness. Physical examination showed only tenderness in the upper abdomen. Laboratory tests showed leukocytosis with relative neutrophilia and hypophosphatemia; other results were normal.

Capecitabine was discontinued. Despite symptomatic therapy (with proton pump inhibitors, antiemetics, parenteral nutrition, analgesia and empiric antibiotics) his clinical condition and laboratory results worsened on the fifth day.

Computed tomography (CT) scan showed a thrombus in the superior mesenteric artery approximately 5 cm distal of the aorta; dilated jejunal, ileal and colonic loops with absence of contrast enhancement in the intestinal wall.

The patient was admitted to the intensive care unit, where he received fluid infusion, vasoactive support with noradrenaline, repeated transfusions of thrombocytes, and fresh frozen plasma; the metabolic acidosis was corrected.

After the patient had been declared stable enough for surgery, a laparotomy was performed and the small bowel, except for the proximal 50 cm of it, along with the right colon were resected despite the rectal tumour. A jejunal-transverse anastomosis was constructed. An attempt at revascularization was not considered due to the clearly necrotic appearance of the affected intestine. Histological examination of the resected gut showed gangrene and multiple thrombi in the vessel walls.

In the intensive care unit he developed sepsis, multi-organ failure (with hemodynamic instability, respiratory insufficiency, hepatic and renal failure, coagulopathy) and fistulae as enteric contents started to leak from the laparotomy wound and through two surgical drains.

The decision was made not to operate but to manage the patient conservatively. The patient’s condition improved; he was hemodynamically stable, he no longer needed oxygen, his organ function was restored and his fever subsided.

On the nineteenth post-operative day, the patient was extubated and he began physiotherapy.

Additional imaging showed enterocutaneous fistulae among the jejunal loops, skin and enterocolic anastomosis, as well as a pancreatic pseudocyst.

The multidisciplinary team composed of radiation therapists, an oncologic surgeon and an anaesthesist specializing in artificial nutrition decided to continue specific oncologic treatment and simultaneously treat gastrointestinal failure with parenteral nutrition.

The rectal carcinoma was treated with short-course pre-operative radiotherapy (5 x 5 Gy) and surgery, during which the fistulae were excised and jejuno-colic re-anastomosis was done. Rectal cancer was treated with total mesorectal excision and permanent colostomy. Post-operatively, the patient developed a paracolic hematoma and a presacral abscess which was drained when he was well enough to be transferred to the regular ward.

The patient remained on home parenteral nutrition (HPN) because of short bowel syndrome. No complications arose during oncologic treatment and the patient is to date in good health and without any signs of recurrence.

## Discussion

Cancer patients face an increased risk of thrombosis as a consequence of their disease and/or its treatment. While the majority of thrombotic events occurring in cancer patients are of venous origin, arterial thrombosis is a well-documented, albeit rarer, entity.

Chemotherapy has been identified as a risk factor for thrombosis and may, depending on the agent, damage the endothelium, induce cytokine release, activate platelets and disrupt the balance between the pro- and anti-thrombotic molecules.^4^

The toxic effects of fluorouracil on the endothelium and the serum concentration of anti-thrombotic molecules have been studied^4^ and most likely apply to capecitabine, a pro-drug of fluorouracil, as well.

Acute mesenteric ischemia (AMI) is an uncommon entity, affecting less than 0.1% of hospitalised patients.^5^ It is most commonly caused by superior mesenteric artery embolism (40–50%) and thrombosis (15–30%).^6^,^7^

With its high sensitivity (96–100%) and specificity (89–92%), CT angiography remains the gold standard for the diagnosis of mesenteric ischemia. 6,7

Treatment decisions are affected by the intraoperative appearance of the bowel. Evidently necrotic bowel loops should be resected, while in any other case, treatment is guided by the principle of arterial reperfusion before intestinal resection is considered, which has in a recent series of three cases proven to be a safe and effective method.^5^

There exists no data about the outcome of intestinal resection in patients with untreated rectal cancer.

Our patient’s surgery resulted in short bowel syndrome (SBS), which is a consequence of a massive anatomical and/or functional loss of intestine, where a reduced small-bowel surface area leads to malabsorption and dehydration. SBS may occur as a consequence of surgery (25%), irradiation/cancer (24–46%), mesenteric vascular disease (15–22%), Crohn’s disease (16–19%) and other benign causes (13–20%).^8^,^9^

The early management of a patient with SBS is that of a critically ill surgical patient, while later, the primary objective is artificial nutritional support at the patient’s home. Enteral intake should be encouraged to minimise dependence on parenteral nutrition.^10^

The patient had type II SBS with a jejunal-colonic anastomosis and from the beginning it was clear that he will remain dependent upon the parenteral supply of adequate energy and nutrients. Despite that the specific oncologic treatment went well with no major complications.

There are three types of intestinal anatomy in SBS, depending on the presence of the colon and ileocecal valve.^11^ Presence of the distal ileum/ileocecal valve is essential to slowing the transit and preventing bacterial overgrowth, while the colon is able to absorb water, electrolytes and fatty acids; both functions serve as a deterrent to the development of diarrhoea, an important cause of malnutrition.^11^

Patients with SBS on parenteral nutrition are at risk of many complications: catheter occlusion and breakage; catheter infections and thrombosis of the central venous access, metabolic disturbances and small bowel bacterial overgrowth.^11^

## Conclusions

Cancer patients face an increased risk of thrombosis as a result of their disease and its treatment. The risks and benefits of aggressive oncological treatment should always be carefully weighed, especially in patients with a good prognosis.

However, with adequate parenteral nutrition and medical support, SBS is not an absolute contraindication for specific oncologic treatment, especially if said treatment is expected to result in cure and/or long-term remission.

Even though the prognosis of patients with bowel necrosis and systemic inflammatory response is dismal, long-term survival is still possible with adequate treatment and management.

## Figures and Tables

**FIGURE 1.**
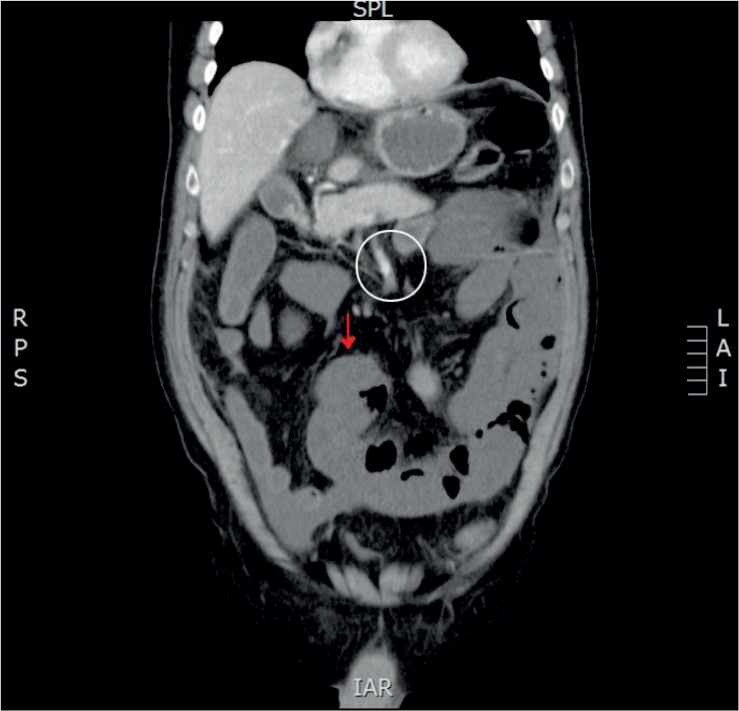
Image of a CT slice which confirmed superior mesenteric artery thrombosis (white circle) and resultant ischemia without contrast enhancement of the bowel wall (red arrow). For comparison there are some bowel loops with contrast enhancement of the bowel wall visible in the upper left part of the image.
